# A Novel Lightweight Polyurethane Composite for Application on Ultra-High-Voltage Insulator Core Filler

**DOI:** 10.3390/polym12112737

**Published:** 2020-11-18

**Authors:** Sizhu Yu, Xiaodong Li, Yage Zhao, Meishuai Zou

**Affiliations:** School of Materials Science and Engineering, Beijing Institute of Technology, Beijing 100081, China; yvsizhu@163.com (S.Y.); bitlxd@bit.edu.cn (X.L.); zhyg0702@163.com (Y.Z.)

**Keywords:** composite materials, thermosets, ultra-high-voltage insulator core filler, polyurethane, lightweight

## Abstract

This study aimed to prepare a new lightweight ultra-high-voltage insulator core filler composite, which can solve the problem of bulkiness. In this study, rigid polyurethane foam pellets with different densities are used as lightweight fillers and polyurethane resins to compound lightweight composite materials. On accounting for working conditions, the density, insulation, heat resistance, water absorption and mechanical properties are tested. The compressive properties of composites are determined by a foam skeleton and a process. Among three kinds of composites, in which the composites with the best comprehensive performance are materials filled with pellets to a density of 0.15g·cm^−3^. The density, surface resistance, volume resistance, leakage current, initial decomposition temperature, water absorption, force, rupture displacement and limiting oxygen index (LOI) of composites are 0.665 g·cm^−3^, 1.17 × 10^14^ Ω, 9.68 × 10^14^ Ω·cm, 0.079 mA, 208 °C, 0.047%, 2262 N, 2.54 mm, and 23.3%, respectively. The ultra-high-voltage insulator core filler in this study can reduce the weight of the solid core insulator crossarm for Ultra-High Voltage (UHV) by 50–75%.

## 1. Introduction

Currently, the composite insulator employed in Ultra-High-Voltage (UHV) transmission systems is the internal insulation solid rod insulator, which is made of epoxy resin and glass fiber and obtained through pultrusion process [1,2,3,4,5,6,7]. The advantages of solid composite insulators are simple structure, excellent mechanical tensile strength, non-breakdown, and excellent stain resistance [8,9]. However, there are also a few disadvantages which are chiefly due to the following: bulkiness, brittleness, low production efficiency, internal core caulking, and interface defects [10,11,12,13,14,15]. The insulating core is filled with an insulating medium as internal insulation, but which does not provide the mechanical strength. Currently, it is mainly composed of epoxy resin [16,17,18,19,20,21].

Polyurethane resin has excellent impact resistance, corrosion resistance, electrical insulation and other advantages [22,23,24]. The most prominent characteristic of polyurethane composite materials is that the performance of the product can be significantly altered through the choice of raw materials and changes in the curing parameters [25,26,27,28]. The development of the material can reduce the weight of the composite insulator crossarm by 50–75% in the UHV environment, which improves the convenience of transportation and also improves the safety of the installation process. The ductile polyurethane resin matrix was adopted to overcome the problem of brittleness existing with epoxy resin. The vacuum casting process of the mandrel in advance can effectively reduce the internal defects of the mandrel, avoids the burning of the mandrel and the problem of cracking caused by the release of intense heat during the process of curing of a large number of resins, and significantly improves the safety performance of the insulator in use.

In this study, by controlling the amount of foaming agent, three types of rigid polyurethane foams with different densities are prepared, which are cut into pellets and combined with the polyurethane resin matrix to prepare lightweight composite materials. Additionally, the density, insulation, heat resistance, water absorption, and mechanical properties of the resin matrix and composites are tested.

## 2. Materials and Methods

### 2.1. Materials

Polymethylene polyphenyl isocyanate (PAPI) was purchased from Yantai Wanhua Polyurethane Co. Ltd., Shandong, China. Castor Oil Polyol (BMY), Polyether Polyol (SA380), Polyether Polyol (MN500), and Diethylene glycol (DEG) were purchased from Shandong Bluestar Dongda Co. Ltd., Shandong, China. Polyester polyol (2680) was supplied by Comite Technology Co. Ltd., Shandong, China. Polyether polyol (4110) was obtained from Shandong Yinuowei New Materials Co. Ltd., Shandong, China. The physical blowing agent (141B) was supplied by Guangzhou Zhonglang Trading Co. Ltd., Guangdong, China. The chemical blowing agent (water) was purchased from Beijing Chemical Works, Beijing, China. Silicone oil foam stabilizer (No. 7, No. 8) was obtained from Meister Chemical Products Co. Ltd., Jiangsu, China. Catalysts (No. 1, 2, 18) were supplied by an air chemical (China) product investment company, Guangdong, China. PAPI, BMY, SA380, MN500, DEG, 2680, 4110, TCPP, No. 7 and No. 8 silicone oil foam stabilizer were chemically pure and the others were analytically pure.

### 2.2. Preparation

All the polyols in this study are dehydrated at 100–110 °C, then cooled to room temperature. The resin matrix was made by one-step method. The polyol and isocyanate were added in beaker at the mass ratio. The mixture hydroxyl compound was consisted of SA380 (7 g), MN500 (40 g), BMY (50 g) and DEG (3 g) per 100 g, which was mixed with 80 g PAPI in a beaker by the one-step method.

The rigid polyurethane foams were also prepared by one-step method. The polyol 2680 and 4110 were mixed at the same mass. Additionally, the mass of foam stabilizer, catalyst, blowing agent and PAPI were added at the mass of 3 g, 0.8 g, 8.5 g and 145 g per 100 g polyol. The mixture was stirred at 500 r·min^−1^ in a beaker. After that, the uniform mixture was poured into the mold immediately and placed for 5 min until the foaming is complete, and then cured at room temperature for 24 h. The foam was removed from the mold and cut into 10 × 10 mm cubes without shuck. Then, the cubes were put into a ball mill and rotated at 500 r·min^−1^ for 5 min. After repeating this step, the rigid polyurethane foam at a density of 0.10 g·cm^−3^, 0.15 g·cm^−3^, and 0.20 g·cm^−3^ can be prepared, respectively.

The polyurethane composite consisted of polyurethane resin and rigid polyurethane foam pellets. Firstly, the rigid polyurethane foam pellets were mixed with uncured resin matrix for the same quality at 53% volume fraction in mold and stirred quickly. Then, the composite was cured at room temperature for 24 h. After that, the uncured liquid resin matrix was poured slowly until 3 cm above all the pellets’ upper surface. The mold was then put into the vacuum oven, evacuated for 8 min and cured 24 h at room temperature. This step was repeated with different density pellets, respectively.

### 2.3. Characterization

#### 2.3.1. Density

The density is defined as the ratio of mass and volume. Therefore, the samples of 60 mm × 60 mm × 2.05 mm were weighted and measured, the ratio of which is the sample density. The 5 samples were subjected to testing, and the average of these was taken as the final result.

#### 2.3.2. Insulation Properties

The insulation properties reflected by volume resistivity (*R*v) and surface resistivity (*R*s), which were tested according to the standard test methods: GB/T 1410-2006 named methods of test for volume resistivity and surface resistivity of solid insulation material by high insulation resistance measuring instrument (ZC-90E type, Shanghai Taiou Electronics Co., Ltd., Shanghai, China).

#### 2.3.3. Thermal Analysis

The glass transition temperature was tested by differential scanning calorimeter (DSC-214, Netzsch Co. Ltd., Freistaat, Germany) in nitrogen atmosphere with purge and protective gas rates of 20 and 60 mL·min^−1^, respectively. The samples, about 5–8 mg, were placed into an aluminum crucible at the rate of 10 °C·min^−1^ from −50 to 250 °C.

The decomposition temperature was tested by thermogravimetric analyzer (TG209F3, Netzsch Co. Ltd., Freistaat, Germany) in nitrogen atmosphere with purge and protective gas rates of 20 and 20 mL·min^−1^, respectively. The samples about 5 mg were placed into porcelain crucibles and heated at the rate of 10 °C·min^−1^ from 30 to 800 °C.

#### 2.3.4. Water Absorption

The water absorption test was carried out by the standard GB/T 1034-2008 plastic-determination water absorption. The samples of 60 mm × 60 mm × 2.05 mm were placed in a dryer at 50 °C for 24 h and weighed and recorded as (*m*_1_). Then, the sample was placed in a container filled with distilled water, and the water temperature was controlled at 23 °C ± 2 °C. After soaking for 24 h, the sample was taken out, the water on the surface of the sample was quickly wiped off with a clean, dry cloth or filter paper, and the sample was weighed again (*m*_2_). The mass fraction of water absorption for each sample was then calculated by (*m*_2_ − *m*_1_)/*m*_1_.

#### 2.3.5. Mechanics

The compression strength test was carried out by the standard GB/T 2567-2008 named test methods for properties of resin casting body. The cylinder sample was Φ10 mm × 25 mm with compression rate at 2 mm·min^−1^. The test is performed 5 times and the average data of that was the final result.

#### 2.3.6. Limiting Oxygen Index (LOI)

The limiting oxygen index was tested by instrument model FTAII (1600) produced by British PL Company (Windsor Court, China). According to the standard (GB/T 2406-93), the sample size is 100.00 mm × 10.00 mm × 4.00 mm, and 10–15 samples are tested in each group to determine the limiting oxygen index.

## 3. Results and Discussion

### 3.1. Lightweight

The lightweight property is measured by density. The difference (*ρ*_d_) between theoretical (*ρ*_cal_) and tested (*ρ*) is used to analyze the bonding interface of matrix and filler. The theoretical density is calculated using the following Equation (1).

(1) $$\rho_{\mathrm{cal}}=\rho_{1}\times V_{1}\%+\rho_{2}\times V_{2}\%$$

where *ρ*_1_ is the density of rigid polyurethane foam pellets, *V*_1_% is the volume fraction of pellets, *ρ*_2_ is the density of the resin matrix, and *V*_2_% is the volume fraction of the resin matrix.

The densities and differences of polyurethane composites are shown in Table 1, and the density of the polyurethane matrix is 1.05 g·cm^−3^. The density of pellets (*ρ*_p_) in this study is 0.10 g·cm^−3^, 0.15 g·cm^−3^, and 0.20 g·cm^−3^, respectively. By comparing the tested and theoretical densities of composites, it can be seen that the tested density is higher than theoretical density. That is because the resin matrix is immersed in the foam holes (could be in an open state) on the surface of pellets, which leads to a decrease in the real volume fraction of the pellet. On the other hand, the defects on the surface of individual pellets lead to the defects of the bubble structure, so that the bubble skeleton is reduced, and the resin enters into defects during the pouring process, which also makes the tested density of composites higher than theoretical density. Moreover, in the process of foaming, the skeleton of some of the pellets is not uniform, resulting in extremely thin foam holes. During the vacuum process of casting the resin, the collapse of the foam structure might lead to the infiltration of the entire pellets, which could be one of the reasons for the tested high density.

As shown in Table 1, the deviation between the tested and theoretical density gradually decreases with an increase in the density of pellets. It is possibly due to that with an increase in the density of pellets, the cells become smaller and smaller, the cell skeleton becomes more and more, and the diameter of the foam cells becomes smaller and smaller. The effective inside volume becomes larger, and thus with an increase in the density of the pellets, the deviation becomes smaller and smaller. Besides, with an increase in the density of the pellets, the pellets become harder and harder, so that the defects of the pellets become less and less, which could also be responsible for the smaller deviation.

### 3.2. Insulation

The surface resistivity (*R*_S_), volume resistivity (*R*_V_), and leakage current (*I*_L_) are used to characterize the insulation properties of composites as shown in Table 2. Additionally, the surface resistivity, volume resistivity, and leakage current of the polyurethane resin matrix are 6.99 × 10^14^ Ω, 5.02 × 10^15^ Ω·cm and 0 mA, respectively.

For application environment, polyurethane foams tend to generate and accumulate charge, and discharge occurs when the amount of charge accumulates to a certain extent. Therefore, the surface resistivity and volume resistivity of the three composite materials are smaller than that of the resin matrix as compared to the polyurethane resin matrix. The insulation of the composite material can be improved by pouring the urethane resin substrate with an excellent insulating property and covering the pellets. As can be seen in Table 2, as the density of pellets increases, the surface resistivity and volume resistivity of composites gradually decreases, indicating that the insulation of the composite gradually deteriorates. It is described that as the density of the urethane pellets increases, the impregnation effect of the urethane resin in the pellets declines, and thus the insulation property becomes weakened. Moreover, studies have shown that a difference in cell morphology leads to different conductivities. When the cell size is small, the cell with a uniform cell distribution has good conductivity. As the foam density increases, the cell size becomes smaller. It is also one of the reasons for the decrease in the resistivity of the composite as the density of the foam beads increases. From the leakage current experiments, it has been noted that as the density of the foam pellets increases, the leakage current of the polyurethane composite increases, which also indicates that the insulation of the polyurethane composite gradually decreases as the density of the filled pellets increases.

### 3.3. Heat Resistance

**Figure 1:**
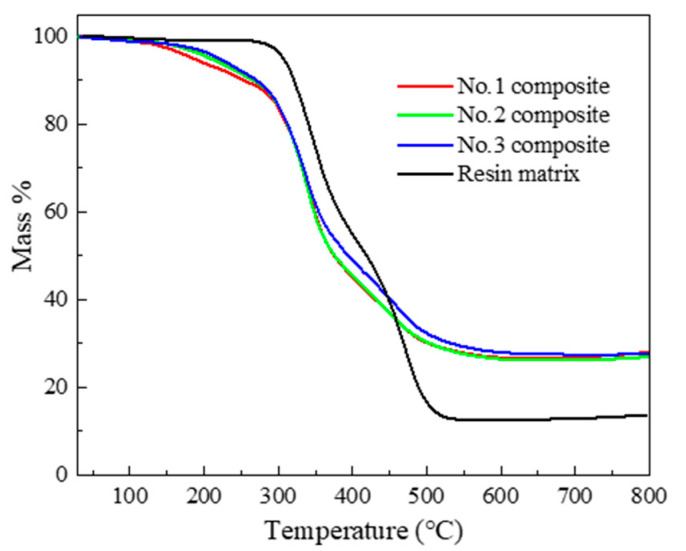
TGA curves of polyurethane composites and resin matrix.

The glass transition temperature and decomposition temperature heat resistance are measured by DSC and TGA, respectively. These properties can determine the application temperature range of composite. Figure 1 shows the TGA curves of the composite, and the Table 3 presents the initial decomposition temperature defined as the temperature that caused the samples to decompose at the mass ratio of 5% (*T*_5%_).

From the DSC test, the glass transition temperature of the resin matrix is 52.3 °C, but the composite has no obvious glass transition. That is because the rigid polyurethane foams usually do not show a glass transition because of the high crosslinking degree. As for the TGA test, the decomposition temperature of the resin matrix is higher than the rigid polyurethane foams. Additionally, with the increase in foam density, the 5% mass decomposition temperature gradually increases. Because the inner diameter of the material becomes smaller, the heat resistance of rigid polyurethane foams is enhanced. The residues of the composite are higher compared to the resin, because the carbon content is higher. Both of the resin and rigid polyurethane foams are decomposed in two stages. The 1st step of decomposition is attributed to the thermal dissociation of urethane groups, and the 2nd step is attributed to the thermal dissociation of the ether or ester group in polyol segments.

### 3.4. Water Resistance

For the application environment of ultra-high-voltage insulator core filler, it must lower hygroscopic. Therefore, the water absorption (*c*_s_) of the composite tested is shown in Table 4. From Table 4, that with the increase in pellet density, the water absorption of the composite does not change distinctly. The water absorption of three types of rigid polyurethane foam composites is almost the same as that of the polyurethane resin matrix. This might be due to the fact that the resin matrix surrounds the pellets during the preparation of the composite and is also surrounded by the resin matrix during the coating of the fixed pellets or the casting process. During the vacuum process, the resin matrix can compact the pellets. From that, there is minimal contact between the foam and the mold, so that water absorption of the composite is substantially equal to the that of matrix.

### 3.5. Mechanical Properties

**Figure 2:**
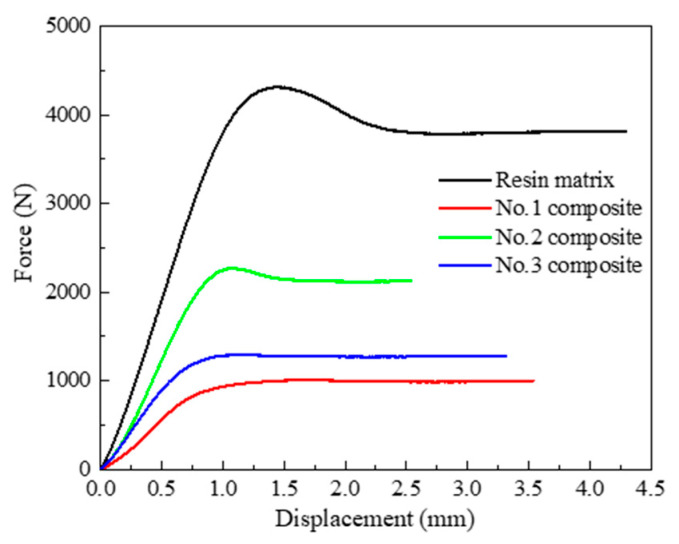
Mechanical curves of polyurethane composites and resin matrix.

For application, ultra-high-voltage insulator core filler must be a certain mechanical support. The force-displacement curves of polyurethane resin matrix and composites are shown in Figure 2 and Table 5. In Figure 2, the pressure on the resin matrix first increases linearly with displacement increase, and then begins to decrease after reaching the peak, and then stabilizes until the compression breaks. The curves of the No.2 composite are the same as that of the resin matrix. The other force-displacement curves are increasing linearly and then stabilize with displacement increases. It is supposed that the inner structure, which has a higher interface bonding force to enhance the mechanical strength, is similar. It shows that, among the three composites, the resin strength performance of the No.2 composite is more obvious. Additionally, the displacement of the composites to the maximum force is basically the same, indicating that in composites, the resin matrix plays the main role of bearing force and the rupture is determined by the force of rigid polyurethane foam pellets and process.

In Table 5, the pressure of the resin matrix is the highest. For composite materials, the force first increases and then decreases with the increase in pellet density, while the change trend of the compression fracture displacement is opposite. Scilicet, the composite filled with pellets at the density of 0.15 g·cm^−3^ has the highest mechanical strength and lowest displacement. However, the other two composites are similar. The moderate foam skeleton and density enable the composites to withstand a higher compressive force, as well as make the plasticity of the material more obvious. Moreover, the resin coats and fills the open pores of pellets with the density of 0.15 g·cm^−3^. Therefore, the No.2 composite has better application value.

## 4. Conclusions

In this study, three kinds of polyurethane composites which filled different density rigid polyurethane foam pellets are prepared, and performance areas such as density, insulation, heat resistance, water absorption and mechanics are tested. As the density of the rigid polyurethane foam filler increases, the density, leakage current and initial decomposition temperature of composites increase, and the heat resistance also increases. Moreover, the volume resistance, surface resistance and maximum force trend increase first and then decrease, and the displacement trend is the opposite. The water absorption of the composite and the resin matrix are similar. All these properties are determined by filler and the process of the composite. The lower-density rigid polyurethane-foam-filled composite material has a thinner foam skeleton and larger cell structure. Simultaneously, during the process, the resin is filled into the open pores on the pellets’ surface, causing resin impregnation, which affects the comprehensive performance of composite. Even if the filler reduces the density of material, it also reduces the other comprehensive properties of the composite, but it is also within the required use range.

Among three kinds of composites, the composites with the best comprehensive performance are materials filled with pellets to a density of 0.15 g·cm^−3^. The density, surface resistance, volume resistance, leakage current, initial decomposition temperature, water absorption, force, rupture displacement and LOI of composites are 0.665 g·cm^−3^, 1.17 × 10^14^ Ω, 9.68 × 10^14^ Ω·cm, 0.079 mA, 208 °C, 0.047%, 2262 N, 2.54 mm, and 23.3%, respectively. Additionally, the LOI of polyurethane resin matrix is 25.3%. The ultra-high-voltage insulator core filler in this study can reduce the weight of the solid core insulator crossarm for UHV by 50–75%, and it has good toughness, compactness and low defect rate. In addition, this composite has excellent adhesion with the fiber-reinforced plastic shell, with excellent electrical insulation performance, low water absorption and long-term water resistance and certain flame resistance performance, and can meet the long-term safety requirements of the composite insulator crossarm in the UHV environment.

## Figures and Tables

**Table 1:** Densities of the polyurethane composites.

No.	*ρ*_p_/g·cm^−3^	*ρ*_t_/g·cm^−3^	*ρ*/g·cm^−3^	*ρ*_d_/g·cm^−3^
1	0.10	0.5465	0.652	0.1055
2	0.15	0.5730	0.665	0.0920
3	0.20	0.5995	0.685	0.0855

**Table 2:** Insulation characteristics of the polyurethane composites.

No.	*ρ*_p_/g·cm^−3^	*R*_S_/Ω	*R*_V_/Ω·cm	*I*_L_/mA
1	0.10	1.51 × 10^14^	1.44 × 10^15^	0.075
2	0.15	1.17 × 10^14^	9.68 × 10^14^	0.079
3	0.20	8.25 × 10^13^	2.26 × 10^14^	0.084
Resin matrix	1.05	6.99 × 10^14^	5.02 × 10^15^	

**Table 3:** Decomposition temperature of the composite and the resin matrix.

No.	*ρ*_p_/g·cm^−3^	*T*_5%_/°C
1	0.10	183.00
2	0.15	208.00
3	0.20	220.00
Resin matrix	1.05	282.00

**Table 4:** Water absorption of polyurethane resin matrix and composites.

No.	*ρ*_p_/g·cm^−3^	*c*_s_ %
1	0.10	0.048
2	0.15	0.047
3	0.20	0.048
Resin matrix	1.05	0.047

**Table 5:** Mechanical properties of polyurethane resin matrix and composites.

No.	*ρ*_p_/g·cm^−3^	Force (N)	*Displacement* (mm)
1	0.10	1005.65	3.54
2	0.15	2266.32	2.54
3	0.20	1292.04	3.27
Resin matrix	1.05	4315.01	4.24
